# Biotransformation of Phenolic Acids in Foods: Pathways, Key Enzymes, and Technological Applications

**DOI:** 10.3390/foods14132187

**Published:** 2025-06-23

**Authors:** Chenxi Lu, Jiayan Zhang, Xiangcheng Zhao, Yuancui Zi, Xiang Xiao

**Affiliations:** School of Food and Biological Engineering, Jiangsu University, Zhenjiang 212013, China

**Keywords:** phenolic acids, biotransformation, enzymatic methods, key enzymes, applications

## Abstract

Phenolic acids, as widely distributed secondary metabolites in plants, possess significant biological activities, such as antioxidant and anti-inflammatory effects. However, their practical applications are limited by low absorption rates and poor bioavailability. Biotransformation technology, with its advantages of strong substrate specificity and mild reaction conditions, has become an effective strategy for the directional modification of phenolic acid molecular structures and the preparation of high-value-added derivatives. Among the various methodologies, enzymatic methods stand out due to their high selectivity and specificity, establishing them as a key approach for phenolic acid biotransformation. The research indicates that coordinated multi-pathway approaches, including decarboxylation, reduction, and hydrolysis, can effectively enhance the efficiency of phenolic acid biotransformation. This review systematically examines the structure and mechanism of action of the key enzymes involved in the phenolic acid biotransformation process. It also proposes innovative pathways and future development directions for existing technologies. Furthermore, it provides an in-depth analysis of the specific application potential of these key enzymes within the food sector. The objective of this review is to furnish a theoretical foundation and technical support for the efficient application of enzymatic methods in phenolic acid biotransformation, thereby accelerating their practical implementation.

## 1. Introduction

In nature, phenolic acids are a class of ubiquitously distributed organic compounds. As important secondary metabolites in plants, their structures contain both phenolic hydroxyl (-OH) and carboxylic acid (-COOH) groups [1,2]. Based on their functional groups, they are classified into hydroxybenzoic acids and hydroxycinnamic acids [3]. The former is based on the benzoic acid structure and contains hydroxyl substitutions on the benzene ring, including *p*-hydroxybenzoic acid, gallic acid, and protocatechuic acid, etc.; the latter is based on the cinnamic acid structure, featuring an additional carbon atom and a double bond on the side chain, along with hydroxyl substitutions on the benzene ring, such as caffeic acid, *p*-coumaric acid, ferulic acid, and sinapic acid, etc. [4].

In plant systems, phenolic acids predominantly exist in two distinct forms: free and conjugated states [5]. Conjugated phenolic acids are typically complexed with carbohydrates, organic acids, or alcohols through esterification or glycosylation reactions, whereas free phenolic acids occur with comparatively lower abundance [6]. Furthermore, phenolic acids exhibit a broad spectrum of bioactivities, including potent antioxidant, anti-inflammatory, anticancer [7,8,9], antimicrobial [10,11], and neuroprotective effects [12,13,14]. These compounds also participate in energy metabolism and biochemical transformations within organisms, significantly modulating lipid and carbohydrate metabolism to regulate blood lipid and glucose levels, thereby maintaining metabolic homeostasis [15,16,17]. Consequently, with advancing research on phenolic acids, their substantial application potential is increasingly recognized in human health promotion, disease prevention, and the development of functional products [18,19,20].

Although phenolic acids exhibit significant biological activities, their absorption efficiency and bioavailability remain relatively low, which limits the exertion of their physiological functions [2]. For example, they are not easily absorbed directly in the gastrointestinal tract and are prone to degradation and metabolic inactivation [21,22,23]. Consequently, enhancing their bioactivity and bioavailability has emerged as a pivotal prerequisite for optimizing their functional performance [24]. Biotransformation technologies, leveraging advantages such as high substrate specificity and mild reaction conditions, have become a strategic approach for the targeted structural modification of phenolic acids and the production of high-value derivatives [25,26]. Current methodologies for phenolic acid transformation primarily encompass chemical, microbial, and enzymatic approaches [27]; however, due to safety considerations in food applications, research has increasingly prioritized microbial and enzymatic methods. Among these, enzymatic biotransformation stands out for its exceptional selectivity and specificity, enabling precise structural modifications to generate derivatives with enhanced bioavailability, making it the focal direction of this review.

Furthermore, significant knowledge gaps persist in the current research concerning the catalytic mechanisms and applications of key enzymes involved in phenolic acid biotransformation, such as phenolic acid decarboxylase, phenolic acid esterase, phenolic acid reductase, and β-glucosidase. To systematically address these gaps, this review first analyzes the primary methods and core pathways of phenolic acid biotransformation, elucidating its significance. Building on this foundation, the review focuses on enzymatic approaches, detailing the molecular structural characteristics and catalytic mechanisms of these key enzymes. It systematically dissects the critical bottlenecks currently hindering research and proposes innovative strategies to overcome them. Furthermore, this review specifically assesses the application potential of these key enzymes within the food industry, with a particular emphasis on analyzing their transformation efficiency and application prospects in specific substrate systems like cereals and fruits. Ultimately, by systematically integrating the multidimensional information presented above, this review aims to provide strategies offering both theoretical guidance and practical applicability. The goal is to promote the efficient utilization and value enhancement of phenolic acid biotransformation technology within the food industry.

## 2. Methods, Pathways, and Significance of Phenolic Acid Biotransformation

The biotransformation of phenolic acids is a current research hotspot, with diverse methods including chemical, microbial, and enzymatic approaches. Key pathways of phenolic acid biotransformation primarily involve decarboxylation, reduction, and hydrolysis, each critically influencing the properties and functions of phenolic acids. The in-depth exploration of the methods, pathways, and significance of phenolic acid biotransformation holds profound importance for unraveling their metabolic mechanisms in organisms, unlocking their potential application value, and advancing interdisciplinary development in related fields.

### 2.1. Methods of Phenolic Acid Biotransformation

#### 2.1.1. Chemical Methods

Chemical methods are technological approaches that directly modify the molecular structure of phenolic acids through chemical reagents or reaction conditions (e.g., acid/base, oxidizing agents, high temperature/pressure). Their core mechanism relies on the cleavage and reconstruction of chemical bonds. Typical operational strategies include: (1) hydrolysis of glycosidic or ester bonds in phenolic acids catalyzed by strong acids or bases to release free phenolic acid monomers [28]; (2) targeted oxidation or reduction of functional groups (e.g., hydroxyl, carboxyl) in phenolic acids using oxidizing or reducing agents, generating derivatives such as quinones or ketones; and (3) selective extraction of phenolic acids via polar organic solvents (e.g., methanol, ethyl acetate), followed by derivatization reactions to enhance chemical stability or detection sensitivity [29].

Although chemical methods demonstrate high efficiency and cost-effectiveness in the conversion of phenolic acids [30], their application in the food industry faces significant limitations. Firstly, the use of chemical reagents such as strong acids/alkalis poses safety hazards, potentially inducing chronic toxicity in humans. Secondly, chemical reactions often suffer from poor selectivity, generating complex and difficult-to-separate byproducts that increase application risks in food products [31]. Moreover, oxidants or extreme pH conditions readily trigger the irreversible degradation of the polycyclic structures of phenolic acids, diminishing their inherent antioxidant activity. Most critically, these methods frequently fail to meet food-grade production standards, thereby running counter to the prevailing trend in the food sector favoring natural constituents.

Given these drawbacks, chemical methods are strictly restricted in the biotransformation of phenolic acids within the food industry. In contrast, microbial and enzymatic methods, due to their mild conditions, high product specificity, and compliance with food application standards, have emerged as mainstream technologies for phenolic acid biotransformation in food industrial applications.

#### 2.1.2. Microbial Methods

Microbial methods primarily utilize the metabolic capabilities of microorganisms for the biotransformation of phenolic acids into value-added compounds [32,33]. Diverse microbial taxa exhibit biotransformation capabilities towards phenolic acids [34,35], including *Lactobacillus* spp. [36], *Saccharomyces* yeasts, and *Aspergillus niger* [37]. For instance, *Saccharomyces cerevisiae* and *Pediococcus acidilactici* BD16 mediated phenolic acid biotransformation during wheatgrass juice fermentation, significantly enhancing its nutritional and functional profiles [38]. Zhou et al. conducted comprehensive investigations into ferulic acid conversion to polyhydroxyalkanoates (PHA), successfully engineering *Pseudomonas putida* KTc9n20 to achieve scalable ferulic acid valorization [39]. Notably, *Streptomyces tunisiensis* DSM 42037 demonstrated exceptional catalytic efficiency by converting free ferulic acid to 4-vinyl guaiacol (14 mg/L) and acetovanillone (12 mg/L), achieving molar biotransform yields of 97% and 83%, respectively [40].

Microbial methods achieve the highly efficient biotransformation of phenolic acids, with specialized strains enabling rapid conversion within short time frames. In addition, microbial methods have significant environmental friendliness and sustainability advantages, which not only can convert different classes of phenolics into more biologically active compounds as a way to change their structure and function, and improve their bioactivity and bioaccessibility [41,42], but also improve the sensory quality of food products [43] and prolong their shelf-life [44]. Therefore, microbial methods have received widespread attention and application in recent years.

#### 2.1.3. Enzymatic Methods

Within phenolic acid biotransformation pathways, multiple enzymes play pivotal catalytic roles, principally including phenolic acid decarboxylase, phenolic acid esterase, phenolic acid reductase, and β-glucosidase. These enzymes cooperatively drive sequential reaction steps through distinct catalytic mechanisms, collectively constituting the enzymatic framework of phenolic acid biotransformation.

As a biotransformation technology, the enzymatic approach employs specific enzymes as biocatalysts to drive phenolic acid biotransformation via enzyme-catalyzed reactions [45,46]. Compared with alternative biotransformation methodologies, enzymatic strategies demonstrate superior selectivity and specificity, enabling the precise catalysis of targeted reactions. Characterized by mild operational conditions typically maintained at ambient temperature and pressure, this approach not only reduces energy consumption but also effectively circumvents potential substrate and product degradation associated with extreme thermal or pressure conditions. However, enzymatic methods face several technical challenges. The intricate processes of enzyme extraction and purification incur substantial costs, while inherent enzyme instability further constrains their industrial-scale implementation [47].

To address these limitations and enhance enzymatic efficiency/stability in phenolic acid biotransformation, researchers have developed advanced technological interventions. Immobilized enzyme technology, for instance, significantly improves thermal stability through enzyme fixation on specialized matrices [48,49,50]. Furthermore, emerging strategies in synthetic biology and metabolic engineering leverage genetic modification techniques to optimize microbial systems for the enhanced expression and catalytic activity of critical enzymes.

The biological transformation technology of phenolic acids has garnered significant attention due to its remarkable application potential in food, pharmaceutical, and cosmetic industries. Through biotechnological approaches such as microbial fermentation and enzymatic catalysis, phenolic acids undergo targeted biotransformation into bioactive derivatives with enhanced antioxidant activity. This process provides a scientific foundation for developing functional foods and nutraceuticals [51]. In comparative analyses between microbial and enzymatic methods, the elucidation of microbial metabolic mechanisms for phenolic acids has established a foundation for the discovery and functional characterization of key enzymes. By isolating key enzymes from microorganisms and optimizing their catalytic function, researchers bypass microbial cultivation complexities to enable direct and efficient phenolic acid biotransformation [27]. Furthermore, technological iterations have accelerated the maturation of enzymatic approaches, exemplified by enzyme engineering and immobilization techniques that substantially improve catalytic efficiency and operational stability.

Consequently, this review emphasizes enzymatic approaches given their dominant status in food processing and high-value product development. Nevertheless, both enzymatic and microbial strategies constitute core technological paradigms for phenolic acid biotransformation, driving value-added utilization through molecular catalysis and systemic metabolic engineering, respectively. These two methodologies exhibit significant functional complementarity in substrate adaptability, product diversity, and process economics [52]. Future research should prioritize their synergistic integration and innovative convergence to enhance the efficiency and application value of phenolic acid biotransformation.

### 2.2. Pathways of Phenolic Acid Biotransformation

**Figure 1:**
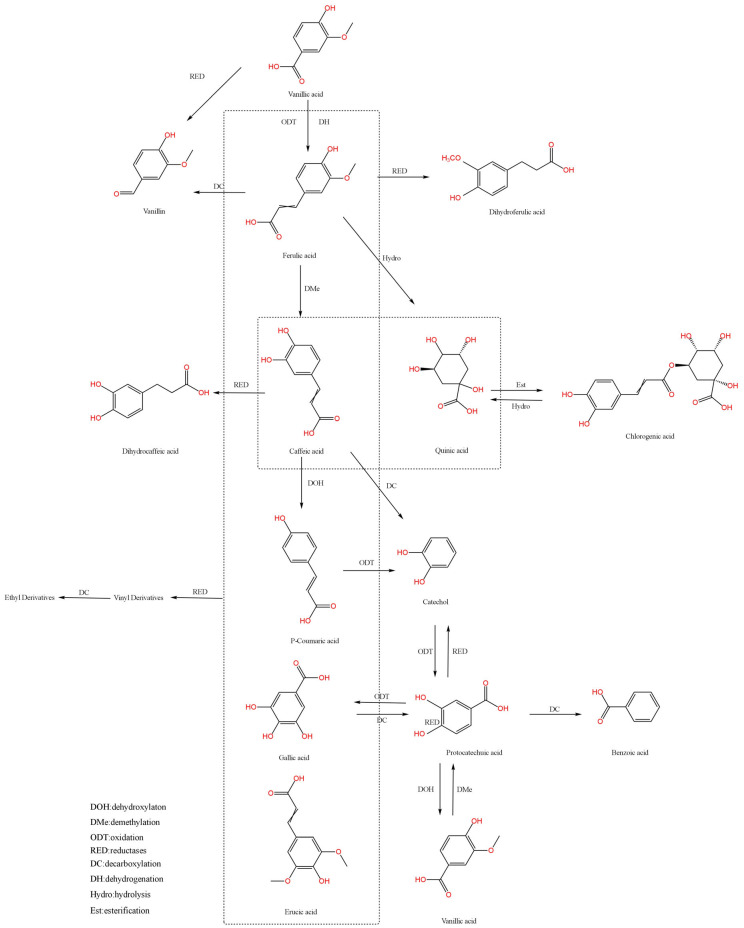
Partial phenolic acid biotransformation pathway diagram.

The biotransformation pathways of phenolic acids play a pivotal role in plant secondary metabolism and microbial degradation processes, involving multi-enzymatic reactions and metabolic network regulation. Decarboxylation, reduction, and hydrolysis constitute the principal biotransformation routes, which demonstrate intricate interconnection through the action of distinct microbial species or enzymatic systems. These interdependent pathways collectively establish a sophisticated metabolic network that governs the biotransformation of phenolic acids [53,54]. The biotransformation pathways of the major phenolic acids are shown in Figure 1.

#### 2.2.1. Decarboxylation

Decarboxylation represents a hallmark pathway in phenolic acid biotransformation, specifically referring to the targeted elimination of the carboxyl group (-COOH) from phenolic acid molecules under the action of biocatalytic systems (microorganisms or specific enzymes), thereby generating phenolic derivatives with distinct physicochemical properties [55]. This process is particularly prominent in the directed conversion of cinnamic acid-type phenolic acids. For instance, phenolic acid decarboxylase (PAD) employs a precise substrate recognition mechanism to catalyze the non-oxidative decarboxylation of typical cinnamic acid derivatives, including ferulic acid, *p*-coumaric acid, caffeic acid, and sinapic acid, yielding aromatic vinyl compounds such as 4-vinylphenol and 4-vinylguaiacol [56]. These products can be used as premium flavoring agents, colorants, or flavor enhancers, commonly found in beverages and condiments. Moreover, due to their potent antioxidant properties, they effectively delay food oxidation.

Furthermore, decarboxylation profoundly alters the chemical structure and bioactivity of phenolic acids. For example, protocatechuic acid is enzymatically decarboxylated to benzoic acid, resulting in modified antioxidant and antimicrobial properties [57,58]. While benzoic acid retains the antioxidant characteristics of its phenolic hydroxyl group, its antioxidant capacity is reduced compared to protocatechuic acid due to the loss of carboxyl group synergism. Conversely, benzoic acid demonstrates significantly enhanced antibacterial activity, achieved through interference with microbial metabolic processes to inhibit bacterial and fungal growth. This dual functionality underpins its widespread application in food preservation and pharmaceutical formulations [59].

#### 2.2.2. Reduction

Reduction serves as a critical pathway in phenolic acid biotransformation, specifically involving the selective hydrogenation or electron transfer of specific functional groups within phenolic acid molecules under biocatalytic systems (microorganisms or reductases), thereby generating reduced-state products such as hydroxyl or methylene derivatives [60]. Microbial systems, exemplified by lactic acid bacteria, catalyze the reduction of caffeic acid and protocatechuic acid to dihydrocaffeic acid and catechol, respectively. This process not only drives chemical transformations of functional groups but also profoundly influences the antioxidant capacity, lipophilicity, and intermolecular interactions of phenolic acids [61].

Further studies reveal that reduction significantly modulates the bioactivity of phenolic acids. Reduced products, such as dihydrocaffeic acid and catechol, exhibit a marked enhancement in antioxidant capacity [62,63]. This phenomenon arises from the ability of reduced phenolic acids to more effectively scavenge free radicals and inhibit oxidative chain reactions. Additionally, reduction can alter the physiological functions of phenolic acids, including strengthening antimicrobial properties or modulating metabolic pathways in biological systems. Such functional shifts highlight the dual role of reduction in both structural modification and bioactivity optimization, underscoring its potential in developing functional foods and bioactive pharmaceuticals.

#### 2.2.3. Hydrolysis

Hydrolysis, as one of the foundational metabolic pathways in phenolic acid biotransformation, is characterized by the enzymatic cleavage of ester bonds (R-O-CO-R’) or glycosidic bonds (C-O-C) in phenolic acid molecules through the action of specific hydrolases (e.g., esterases, glycosidases), yielding low-molecular-weight phenolic compounds and glycosyl ligands [64].

For instance, chlorogenic acid undergoes hydrolysis catalyzed by esterases to generate caffeic acid and quinic acid [65]. This process not only modifies the chemical architecture of chlorogenic acid but also profoundly impacts its bioactivity and functional properties [66]. Specifically, caffeic acid exhibits potent antioxidant capacity, capable of effectively scavenging free radicals and suppressing oxidative reactions. In contrast, quinic acid demonstrates distinct biological functionalities, such as modulating gut microbiota composition, thereby highlighting its potential applications in food and pharmaceutical industries [67].

The biotransformation of phenolic acids primarily occurs through three core metabolic pathways: decarboxylation, reduction, and hydrolysis. These pathways collectively construct a multi-layered and multi-target metabolic network, thereby expanding the structural diversity of phenolic compounds and enabling the precise modulation of their functional activities. By delving into the mechanisms and regulatory factors of these pathways, it becomes possible to harness biotransformation technologies for the production of phenolic acid derivatives with specific functionalities. This approach not only advances the understanding of phenolic acid metabolism but also provides novel products and raw materials for industries such as food, pharmaceuticals, and fragrances.

### 2.3. Significance of Phenolic Acid Biotransformation

In contemporary scientific research and practical applications, phenolic acid biotransformation demonstrates multifaceted significance across multiple dimensions.

From the perspective of resource utilization, phenolic acid biotransformation technology enhances the value of natural products through enzyme-catalyzed specificity. For instance, lignin-derived phenolic acids are microbially decarboxylated into high-value-added flavor compounds such as vanillin, which also serve as critical precursors for synthesizing pharmaceuticals [68]. This approach not only aligns with sustainable development principles but also meets consumer preferences for natural products. During lactic acid bacteria fermentation, phenolic acid decarboxylases convert ferulic acid into vinyl derivatives [69]. These compounds not only contribute to the characteristic flavors of fermented foods like wine and kimchi but also regulate microbial phenotypes [70], such as biofilm formation, by generating quorum-sensing signaling molecules [71]. Notably, the transformed roots of *Rhaponticum carthamoides* have been identified as an effective source of caffeoylquinic acid derivatives (CQAs) [72,73], which exhibit health-promoting bioactive properties [74]. This dual-function process improves soil microenvironments while yielding functional molecules with health benefits. Additionally, breakthroughs in environmental remediation have been achieved using phenolic acid decarboxylases (e.g., BsPAD from *Bacillus subtilis*) [75], which enable the green biotransformation of phenolic pollutants into environmentally benign products under mild reaction conditions [76].

Phenolic acid biotransformation research has emerged as a pivotal driver of biotechnological innovation, significantly advancing fields such as biocatalysis and enzyme engineering [77]. Through sustained research, researchers have developed high-efficiency biocatalysts, optimized enzymatic activity, and screened novel microbial strains enabling efficient phenolic acid biotransformation. These innovations have significantly enhanced biotransformation efficiency, product yield, and cost-efficiency, delivering robust technical foundations for industrial-scale implementation. Crucially, phenolic acid biotransformation constitutes a pivotal resource utilization strategy, where key enzymes govern pathway specificity, catalytic efficiency, and the bioactivity enhancement of final products. In-depth investigations into the structural characteristics, mechanistic principles, and practical applications of these enzymes are therefore essential for advancing the sustainable and high-value utilization of phenolic acid resources.

## 3. Structure and Mechanisms of Key Enzymes in Phenolic Acid Biotransformation

In the study of key enzymes involved in phenolic acid biotransformation processes, we focus on elucidating the pivotal roles of multiple enzymes within these pathways. These enzymes—including phenolic acid decarboxylase, phenolic acid reductase, phenolic acid esterase, β-glucosidase, and various auxiliary enzymes—catalyze distinct steps in phenolic acid biotransformation at different functional levels. The elucidation of their structural characteristics and mechanistic principles holds profound significance for advancing the understanding of phenolic acid metabolic networks, optimizing related bioprocesses, and developing novel biotechnological applications. By dissecting their structure–function relationships and catalytic dynamics, this research provides critical insights into the molecular strategies driving enzymatic specificity and efficiency, thereby laying a foundation for the rational design of biocatalysts in synthetic biology and industrial biotechnology.

### 3.1. Phenolic Acid Decarboxylase

#### 3.1.1. Structure of Phenolic Acid Decarboxylase

Phenolic acid decarboxylase (PAD, EC 4.1.1.102) represents a crucial enzyme category that catalyzes the decarboxylation of phenolic acid derivatives [78,79]. This enzyme exhibits substrate specificity in removing the carboxyl group (–COOH) to generate volatile 4-vinylphenolic derivatives, including 4-vinylguaiacol and 4-vinylphenol [80]. Widely distributed across microbial and plant systems, PAD plays significant roles in food fermentation processes and natural product biosynthesis.

The structural architecture of PAD demonstrates precise adaptation to its catalytic functionality, where molecular conformation and critical domain organization directly govern substrate recognition, reaction efficiency, and environmental adaptability. Typically existing as homodimers or homotetramers [81], PAD belongs to the α/β-hydrolase superfamily. Its core structure features a characteristic “α/β-barrel” folding pattern, comprising parallel or mixed β-sheets flanked by surrounding α-helices. This configuration creates a stable hydrophobic microenvironment essential for substrate accommodation and catalytic activity.

#### 3.1.2. Mechanisms of Phenolic Acid Decarboxylase

The current understanding of the PAD catalytic mechanism remains limited, with the reported studies primarily attributing the decarboxylation process to synergistic interactions among conserved amino acid residues (e.g., Glu134, Asn23, Tyr27) within the active site. The reaction proceeds through four distinct stages: Firstly, the phenolic hydroxyl group of the substrate undergoes protonation mediated by a glutamate residue. Subsequently, electronic rearrangement induces the formation of a nucleophilic center at the carbon adjacent to the carboxyl group. Concurrently, asparagine (Asn) stabilizes a water molecule via hydrogen-bonding networks, serving as a proton donor to facilitate the generation of a quinoid transition-state intermediate. Finally, CO_2_ is released, yielding volatile 4-vinylphenolic derivatives as the reaction product [82,83,84].

The research confirms that acid–base catalysis dominates the PAD catalytic core, with its active site containing a hydrophobic cavity critical for substrate binding and reaction efficiency [82]. Structural analyses further reveal functional differentiation between the enzyme’s terminal extensions: The C-terminal extension enhances acid tolerance, while the N-terminal extension appears to optimize substrate binding in alkaline environments through improved cavity interactions and structural stabilization [85]. Additionally, Huang et al. [86] identified a notable regulatory effect of reducing agents, including dithiothreitol (DTT), 2-mercaptoethanol, cysteine, and homocysteine, which significantly accelerated ferulic acid decarboxylation activity in both native and recombinant CgPAD. Intriguingly, these agents exhibited no detectable influence on *p*-coumaric acid decarboxylation activity, suggesting the substrate-specific modulation of enzymatic function by redox-sensitive molecular interactions.

### 3.2. Phenolic Acid Esterase

#### 3.2.1. Structure of Phenolic Acid Esterase

Phenolic acid esterase (ESTR, EC 3.1.1), as a crucial member of the carboxylesterase family, plays a pivotal catalytic role in biological metabolic processes. This enzyme participates in diverse biotransformation pathways through the specific hydrolysis of ester bonds in phenolic acid derivatives. Widely distributed in plants, microorganisms, and animals, ESTR demonstrates significant biological relevance and industrial application potential [87].

Structurally, ESTR exhibits the characteristic α/β hydrolase fold configuration, with its active center formed by the conserved Ser-His-Asp catalytic triad [88,89]. The serine residue (Ser) acts as a nucleophile to directly attack the carbonyl carbon of the ester bond, while histidine (His) functions as a general base to activate the serine hydroxyl group and mediate proton transfer. Aspartate (Asp) stabilizes the protonation state of histidine, thereby maintaining the catalytic microenvironment. This distinctive structural architecture endows ESTR with remarkable hydrolytic efficiency, enabling its specific activity toward hydroxycinnamate esters and facilitating critical metabolic reactions in biological systems [90].

#### 3.2.2. Mechanisms of Phenolic Acid Esterase

In terms of the catalytic mechanism, ESTR employs the core Ser-His-Asp catalytic triad to execute a two-step nucleophilic process. Initially, the serine residue in the active site initiates a nucleophilic attack on the ester bond’s carbonyl carbon, forming a tetrahedral transition state intermediate. Subsequently, the acyl-enzyme intermediate undergoes hydrolytic deacylation, achieving efficient substrate conversion.

This catalytic mechanism is exemplified in the wolfberry fermentation system. The highly efficient ESTR system of *Lactobacillus plantarum* NCU137 specifically hydrolyzes chlorogenic acid into quinic acid and caffeic acid [91,92]. This biotransformation process not only enhances the flavor profile of fermented products but also yields phenolic acid derivatives exhibiting marked antioxidant activity. These findings provide valuable insights for functional food development.

### 3.3. Phenolic Acid Reductase

#### 3.3.1. Structure of Phenolic Acid Reductase

Phenolic acid reductase (PAR, EC 1.3.1) is a class of enzymes capable of catalyzing the reduction of phenolic acid derivatives. As a critical oxidoreductase ubiquitously distributed in microorganisms and plants, its primary function lies in the specific catalysis of phenolic acid compounds (e.g., ferulic acid, *p*-coumaric acid, protocatechuic acid), thereby altering their chemical properties and biological activities [93].

The overall structure of PAR typically comprises one or more cofactor-binding sites, such as flavin adenine dinucleotide (FAD) or flavin mononucleotide (FMN), which play a pivotal role in electron transfer during catalysis [94]. The enzyme’s active site harbors key amino acid residues, including histidine (His) and cysteine (Cys), which stabilize substrates through hydrogen bonding or covalent interactions, facilitating catalytic efficiency. Furthermore, the enzyme surface features a substrate-binding pocket whose size and shape determine substrate specificity. This pocket is lined with hydrophobic and polar residues that engage the substrate via hydrogen bonds, hydrophobic interactions, and electrostatic forces, ensuring precise substrate positioning for the catalytic reaction.

#### 3.3.2. Mechanisms of Phenolic Acid Reductase

Regarding the catalytic mechanism, the substrate initially binds to the enzyme’s active site, where key amino acid residues form hydrogen bonds or other stabilizing interactions to ensure proper substrate orientation. Subsequently, the cofactors (e.g., FAD or FMN) within the active site accept electrons from the substrate, generating transient intermediate species. These intermediates ultimately transfer electrons to terminal acceptors such as NAD(P)^+^, thereby completing the reduction process [88].

In *Lactiplantibacillus plantarum*, the hydroxycinnamic acid reductase HcrB has been demonstrated to reduce hydroxycinnamic acids to vinylphenol derivatives [60]. Concurrently, the enzymatic system VrpA facilitates the conversion of vinyl derivatives to ethyl derivatives via a decarboxylation–reduction coupled reaction [95].

### 3.4. β-Glucosidase

#### 3.4.1. Structure of β-Glucosidase

β-Glucosidase (BGL, EC 3.2.1.21) belongs to the glycosidase family within the hydrolase superfamily [96], catalyzing the hydrolysis of glycosidic bonds linking terminal non-reducing sugar residues to oligosaccharides or aromatic/alkyl aglycones [97,98].

Structurally, β-glucosidases typically adopt a conserved (β/α)_8_ barrel catalytic domain, characteristic of glycoside hydrolase families such as GH1 and GH3. The active site harbors two critical acidic amino acid residues (e.g., glutamic acid [Glu] and aspartic acid [Asp]), which function as a general acid and general base, respectively. Substrate binding involves hydrogen bonding and hydrophobic interactions between the glucosyl moiety of the β-glycosidic bond and conserved residues (e.g., tryptophan, tyrosine) in the active site, ensuring the stereospecific recognition of the β-configuration. Concurrently, the aglycone moiety is stabilized within a hydrophobic pocket [99].

#### 3.4.2. Mechanisms of β-Glucosidase

The catalytic mechanism proceeds via two sequential steps: First, the general acid residue donates a proton (H^+^) to the glycosidic oxygen, inducing protonation. This weakens the glycosidic bond, leading to its cleavage and the formation of a glycosyl-enzyme covalent intermediate, with the concomitant release of the aglycone. Next, the general base residue activates a water molecule by abstracting a proton, generating a nucleophilic hydroxide ion (OH^−^). This ion attacks the anomeric carbon of the glycosyl-enzyme intermediate, releasing β-D-glucose and restoring the enzyme’s active state [88].

Variations in catalytic residues or cofactor requirements exist among β-glucosidases from different origins. For instance, GH3 family member TtBgl3 (from *Trametes trogii* S0301) exhibits exceptional phenolic compound deglycosylation activity, positioning it as a promising green biocatalyst for synthesizing bioactive glycosides in pharmaceutical and biotechnological applications [100].

### 3.5. Other Enzymes Involved in the Biotransformation of Phenolic Acids

The biotransformation of phenolic acid compounds involves a sophisticated enzymatic network beyond the well-characterized enzymes such as phenolic acid decarboxylases, phenolic acid reductases, phenolic acid esterases, and β-glucosidases. Emerging evidence highlights the indispensable roles of several understudied enzymes in regulating critical steps of phenolic acid metabolic pathways.

In lignification pathways, Zea mays CINNAMYL ALCOHOL DEHYDROGENASE 2 (ZmCAD2), a cinnamyl alcohol dehydrogenase, has been demonstrated to participate in the reduction of hydroxycinnamaldehydes. A mutant analysis revealed that ZmCAD2 deficiency leads to the abnormal accumulation of hydroxycinnamaldehydes, which are partially converted into (dihydro) ferulic acid and sinapic acid derivatives, thereby highlighting its crucial role in maintaining the homeostasis of lignin precursors [101].

Furthermore, the cinnamate-4-hydroxylase AnanC4H, identified from Anomoloma albolutescens, serves as a novel biocatalyst for phenolic acid hydroxylation by specifically catalyzing the conversion of trans-cinnamic acid to *p*-coumaric acid [102].

In the metabolic processes of the filamentous fungus *Aspergillus niger*, two critical enzymes involved in this pathway have been identified through previous research: para-hydroxybenzoate hydroxylase (phhA) and protocatechuate 3,4-dioxygenase (prcA). Significantly, a protocatechuic acid hydroxylase (PhyA) has been successfully characterized, revealing a novel alternative metabolic pathway capable of converting protocatechuic acid into hydroxyhydroquinone [57].

**Figure 2:**
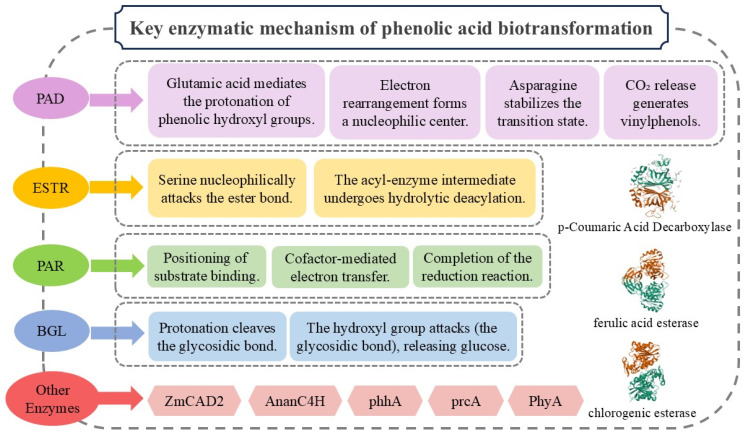
Key enzymatic mechanism of phenolic acid biotransformation.

These discoveries not only enhance the theoretical framework of enzymatic systems associated with phenolic acid biotransformation, but more importantly, through the identification of these key enzymes, provide innovative strategies for the microbial production of high-value phenolic acid derivatives. Figure 2 summarizes the mechanisms of key enzymes in phenolic acid biotransformation [103,104].

## 4. Multidimensional Innovation and Development in Key Enzyme Research

Although existing studies have preliminarily established application frameworks for phenolic acid-biotransforming enzymes, substantial technological breakthroughs remain imperative in the following critical dimensions.

### 4.1. Systematic Enhancement of Key Enzyme Molecular Performance

The application of key enzymes in phenolic acid biotransformation faces three primary technological bottlenecks: limited catalytic efficiency, insufficient environmental tolerance, and a narrow substrate spectrum. Addressing these challenges requires synergistic approaches across two dimensions: enzyme molecular engineering and immobilization technology.

Recent studies have elucidated the molecular mechanisms underlying enzyme structure-function relationships. Strategic modifications to the C-terminal and N-terminal regions of phenylacrylic acid decarboxylase revealed that C-terminal extension enhances acid resistance, while N-terminal extension improves alkali resistance and thermal stability [85]. Leveraging computer-aided molecular design, advanced artificial engineering techniques have successfully identified critical amino acid residues governing PAD thermostability, thereby establishing a theoretical foundation for directed evolution [105,106].

In the field of immobilization technology, an innovative reversibly soluble polymer-based dual-enzyme co-immobilization system (feruloyl esterase and xylanase) has been developed. Through the systematic characterization of enzymatic properties and catalytic conditions, this system demonstrated efficient ferulic acid production from wheat bran hydrolysis using co-immobilized enzymes [49,107]. This technological advancement lays the groundwork for engineering multi-enzyme cascade reaction systems in industrial applications [108].

### 4.2. In-Depth Exploration and Functional Development of Enzyme Resources

Current research on phenolic acid biotransformation enzymes remains limited in scope, with numerous unidentified enzymes still awaiting characterization in this metabolic pathway. Notably, a breakthrough study identified the first basidiomycetous ferulic acid decarboxylase (designated ScoFAD) from *Schizophyllum commune*, which was heterologously expressed in *Komagataella phaffii*. This enzyme demonstrates distinctive catalytic properties, including a molecular mass of 21 kDa and optimal activity at pH 5.5 and 35 °C. Remarkably, it exhibits a catalytic efficiency (kcat/KM) of 4779 L·s^−1^·mmol^−1^, surpassing the second-best documented enzyme by over 50-fold [109].

Furthermore, integrating metagenomic and functional transcriptomic technologies can overcome cultivation bottlenecks, enabling the discovery of novel phenolic acid-biotransforming enzymes from extreme environment microorganisms or unculturable microbes, thereby expanding their application scopes.

### 4.3. Construction of Multi-Enzyme Cascade Systems

In recent years, the biotransformation strategy for phenolic acids based on multi-enzyme cascade catalytic systems has garnered significant attention due to its high efficiency and sustainability. Myrtollari K et al. developed an innovative one-pot two-step cascade system: Initially, BsPAD derived from *Bacillus subtilis* catalyzes the decarboxylation of phenolic acid substrates such as coumaric acid, caffeic acid, ferulic acid, and kaempferol acid. Subsequently, resveratrol O-methyltransferase (VvROMT) facilitates specific O-methyl transfer of the resulting phenolic styrene intermediates [110]. Notably, experimental validation by Petermeier et al. demonstrated complete substrate conversion within 3 h in high-concentration (400 g·L^−1^) PAD-mediated continuous cascade reactions, highlighting the system’s potential for biomanufacturing applications [111].

Nevertheless, the industrial implementation of multi-enzyme cascade systems still faces critical challenges. Significant disparities in optimal reaction conditions among cascade enzymes often hinder the synchronized stabilization of enzymatic activities across sequential steps. Furthermore, the dynamic accumulation of intermediate products may induce substrate/product inhibition effects, leading to process stagnation in cascade reactions. Addressing these bottlenecks can be achieved through the development of AI-driven cascade designs to enable highly efficient and precise regulation [112,113].

### 4.4. Synthetic Biology-Driven Enzyme Function Enhancement

Based on synthetic biology theory, microbial metabolic network reconstruction technology has emerged as a pivotal approach for enhancing phenolic acid biotransformation efficiency [111]. Through the synergistic integration of CRISPR-Cas9 gene editing technology and metabolic engineering [114], the following breakthroughs have been achieved: Targeted cloning of gene sequences encoding key enzymes for phenolic acid biotransformation enables high-efficiency expression of target genes [115]. Furthermore, the implementation of fusion protein engineering strategies significantly improves the biocatalytic efficiency and catalytic activity of these critical enzymes.

With the iterative advancement of protein engineering technologies, the establishment of heterologous expression systems and molecular modification of key enzyme proteins have been achieved [116,117]. Rational design strategies have been employed to optimize their substrate specificity and catalytic kinetic parameters, thereby effectively expanding their versatile applications in biocatalytic processes across multiple scenarios.

### 4.5. Precision Design of Microbial Cell Factories

Although microbial cell factories exhibit substantial potential in phenolic acid biotransformation, they still face challenges such as substrate adaptability limitations and metabolic network complexity. By integrating synthetic biology and metabolic engineering technologies, microbial cell factories with high-efficiency substrate conversion capabilities can be systematically engineered [118], enabling the large-scale production of phenolic acids. For instance, Chen et al. developed a microbial platform for high-level production of caffeic acid and ferulic acid, critical precursors for numerous pharmaceutical molecules [119]. Similarly, Lubbers RJM et al. achieved biotransformation pathway engineering in *Aspergillus niger* cell factories to produce protocatechuic acid [57].

**Figure 3:**
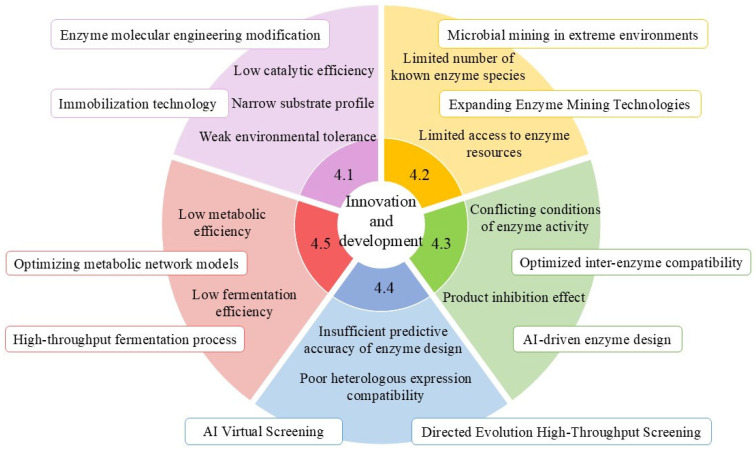
Multidimensional innovation and development of key enzymes in phenolic acid biotransformation applications.

Figure 3 comprehensively illustrates the existing problems and challenges, revealing that current technological frontiers exhibit a distinct trend toward interdisciplinary convergence: artificial intelligence (AI)-driven enzyme virtual screening [120,121], microfluidic chip-assisted high-throughput evolution platforms, and metabolomics-guided dynamic pathway regulation technologies are collectively advancing phenolic acid conversion technologies toward precision and intelligent evolution.

## 5. Applications of Key Enzymes in Phenolic Acid Biotransformation in the Food Industry

Phenolic acids account for nearly one-third of the total dietary phenolic compounds in cereals and fruits [122]. However, significant differences exist in the types, content, and existing forms of phenolic acids among cereals, fruits, and vegetables, stemming from plant-specific metabolic pathways and tissue functions. Key enzymes involved in the biotransformation of phenolic acids exhibit broad application potential in the food industry, particularly in cereals and fruits.

### 5.1. Applications in Cereals

The forms and biotransformation pathways of phenolic acids in cereals significantly influence food quality. In cereals, phenolic acids predominantly exist in bound forms, with ferulic acid being the most abundant component, accounting for 70–90% of total phenolic acids [123,124,125]. In wheat, rice, and maize, ferulic acid is cross-linked with arabinoxylans via ester bonds in the bran layers, forming structural components of cell walls [118,126,127], while oats and barley contain higher proportions of *p*-coumaric acid [128]. Additionally, processing methods such as germination or fermentation can activate endogenous enzymes to release bound phenolic acids [117,129], thereby enhancing their antioxidant activity. Table 1 summarizes key enzymes involved in the biotransformation of phenolic acids and their applications in cereals.

Recent mechanistic studies revealed that *Monascus* spp. fermentation achieves synergistic catalysis through cellulase-decarboxylase coupling [130,131,132], enabling the cascade conversion of oat phenolic acids: Ferulic/*p*-coumaric acids undergo sequential decarboxylation-hydroxylation to generate caffeic acid, which subsequently combines with quinic acid to synthesize chlorogenic acid [133]. In Awamori sake brewing, the AlPAD esterase from *Aspergillus niger* specifically hydrolyzes ferulic acid ester bonds in rice cell walls. The resultant decarboxylation product 4-vinylguaiacol (4-VG) is subsequently oxidized to vanillin, with this conversion pathway being rigorously validated through isotopic tracer techniques [134].

**Table 1:** Applications of key enzymes for phenolic acid biotransformation in cereals.

Enzyme	Original Phenolics	Transformed Products	Microorganism	Food Matrix	Role	Reference
Cellulase	Ferulic acid and *p*-coumaric acid	Caffeic acid	*Monascus anka* GIM 3.592	Oatmeal	Releases bound phenolic acids and improves antioxidant properties	[133]
AlPAD	Ferulic acid	4-Vinylguaiacol	*Aspergillus luchuensis*	Steamed rice	Development of characteristic flavors	[134]
Lp_0796, Est_1092	Ferulic acid	Dihydroferulic acid	*Lactobacillus plantarum* TMW1.460	Whole wheat flour	Altering the sensory properties of food	[135]
par1, par2, estR, pad	Hydroxycinnamic acid	Vinyl Derivatives	*Furfurilactobacillus milii* FUA3583	Sorghum	Improvement in antimicrobial activity	[136]
PCD	Ferulic acid	Vinyl guaiacol Ethyl guaiacol Dihydroferulic acid	*Lactobacillus plantarum*	Wheat sourdough	Affects the nutritional content and texture of bread	[137]
Cellulase, hydrolytic enzyme, and β-glucosidase	*p*-Coumaric acid and caffeic acid	Chlorogenic acid	*Monascus anka* GIM 3.592, *Saccharomyces cerevisiae* GIM 2.139, and *Bacillus subtilis* 784	*Avena sativa* L.	Improvement in functional properties of cereal products	[138]
-	Soluble protocatechuic acid and soluble vanillic acid	Alcohol-soluble protocatechuic acid and alcohol-soluble vanillic acid	*Aspergillus oryzae* 6001, *Aspergillus oryzae* 6020, *Aspergillus sojae* 700, and *Aspergillus luchuensis* 8035	Rice	Increased antioxidant activity	[139]
Hydroxycinnamic acid reductase	Caffeic acid and ferulic acid	Dihydrocaffeic acid and dihydroferulic acid	*Candida milleri*, *Lactobacillus brevis*, and *Lactobacillus plantarum*	Whole wheat and rye	Able to regulate blood lipid and blood sugar levels	[140]
-	Ferulic acid	4-Vinylguaiacol	*Streptomyces tunisiensis* DSM 42037	Barley bran	Potential applications in the food, pharmaceutical, and cosmetic industries	[40]
-	Ferulic acid and caffeic acid	Dihydroferulic acid and dihydrocaffeic acid	*Lactobacillus plantarum* DSMZ 13890	Rye	Regulates gut and host health	[141]

- Indicates no specific enzyme.

Notably, microbial metabolic diversity studies have identified strain-specific conversion patterns: The *Lactiplantibacillus plantarum* TMW1.460 mutant preferentially accumulates dihydroferulic acid during whole wheat fermentation [136], whereas *Fructilactobacillus milii* FUA3583 predominantly mediates the reductive metabolism of hydroxycinnamic acids in sorghum systems [136]. These differential metabolic profiles are intrinsically associated with strain-specific substrate specificity and differential expression levels of oxidoreductase systems, thereby establishing a molecular foundation for targeted regulation of flavor profiles and functional characteristics in cereal-based foods [142].

Therefore, the biotransformation of phenolic acids in cereals releases free phenolic acids with higher activity, enhancing antioxidant capacity and absorption rates. Simultaneously, it degrades anti-nutritional factors, improves food flavor and texture, and generates functional components such as antimicrobial or anti-inflammatory agents, demonstrating significant application potential in food processing [143].

### 5.2. Applications in Fruits

Fruits serve as a primary dietary source of phenolic acids, with hydroxycinnamic acids dominating their phenolic profiles, particularly caffeic acid and chlorogenic acid. For instance, berries such as blueberries and cherries are rich in both free and conjugated caffeic acid derivatives [144]. Furthermore, hydroxybenzoic acids like ellagic acid are notably abundant in red fruits such as strawberries, raspberries, and blackberries, where they often bind with tannins to form macromolecular complexes [145]. Total phenolic acid content in fruits varies significantly with cultivar and maturity; for example, phenolic levels decline during the ripening of *Pistacia lentiscus* L. fruits.

Phenolic acid decarboxylases serve as pivotal biocatalysts in fruit fermentation processes, exerting substrate-inducible catalytic properties to convert free phenolic acids into characteristic aromatic compounds such as 4-vinyl/ethylphenol derivatives, which critically contribute to the spicy and herbal flavor profiles of wines [146]. Microbial-derived PDCs from *Bacillus subtilis* and *Lactiplantibacillus plantarum* exhibit substrate-specific decarboxylation activity towards free phenolic acids [147]. Simultaneously, engineered phenolic acid decarboxylase whole-cell biocatalytic systems utilizing surface-displayed *Saccharomyces cerevisiae* enable efficient hydroxycinnamic acid decarboxylation, achieving targeted synthesis of vinylphenolic pyranoanthocyanins in blueberry wine [148,149].

In fruit juice processing systems, phenolic acid reductases play essential roles in quality modulation through reductive modification. During the lactic acid fermentation of cherry juice, these enzymes catalyze the biotransformation of caffeic acid to dihydrocaffeic acid, while mediating the transformation of *p*-coumaric acid into 4-ethylphenol and phenyllactic acid [150]. The resultant metabolites not only impart distinctive aromatic characteristics to fermented products but also improve product stability through redox equilibrium regulation [41,151,152].

Modern fruit processing has established systematic enzymatic synergy strategies: In winemaking, decarboxylase-reductase cascade reactions construct comprehensive phenolic acid conversion networks. Apple juice production employs esterase–reductase combinatorial processes to achieve simultaneous phenolic acid biotransformation and enzymatic browning inhibition [153,154,155]. Table 2 systematically summarizes the functional applications of key phenolic acid-converting enzymes in fruit processing systems.

### 5.3. Applications in Other Foods

The key enzymes involved in phenolic acid biotransformation demonstrate multidimensional value in vegetable processing. In fermentation applications (e.g., pickle production), phenolic acid decarboxylase catalyzes the decarboxylation of phenolic acids to generate 4-vinyl derivatives. This conversion not only imparts distinctive flavor profiles but also enhances the overall sensory quality [162]. For instance, the *Lp. plantarum* TMW1.460 mutant strain specifically accumulates dihydroferulic acid and dihydrocaffeic acid during broccoli fermentation [163], providing novel insights for the targeted regulation of fermentation products [135]. In preservation technology, phenolic acid reductase effectively maintains vegetable sensory attributes and extends shelf life by inhibiting phenolic acid oxidation. During deep processing, ESTR improves both the antioxidant capacity and nutritional value of products through the liberation of bound phenolic acids [164,165].

The biotransformation of rapeseed meal (CM), an oil processing byproduct, highlights the synergistic effects of enzymatic systems. ESTR and BGL activate precursor substances to release antimicrobial phenolics, while the decarboxylase system converts substrates such as ferulic acid and caffeic acid into potentially valuable products like 4-vinylcatechol [166]. These findings establish crucial technical pathways for the high-value utilization of agricultural byproducts [167]. Furthermore, when heterologously expressed, caffeic acid O-methyltransferase derived from neem fruit (NCOMT) catalyzes the efficient biotransformation of caffeic acid into pharmaceutically significant ferulic acid. This establishes novel routes for the biosynthesis of high-value natural compounds [168]. The natural enzyme NlePAD converts sinapic acid into canolol (2,6-dimethoxy-4-vinylphenol) through non-oxidative decarboxylation, which exhibits stronger antioxidant activity. This product demonstrates significant development potential in functional foods and cosmetics industries [169].

Current research reveals that both food matrix characteristics and critical enzymes influence phenolic acid biotransformation. Table 3 primarily lists the application scenarios of key enzymes for phenolic acid biotransformation in various other food products. The variability in phenolic acid composition and biotransformation pathways across food systems necessitates the development of customized enzymatic strategies. These discoveries not only advance the theoretical understanding of phenolic acid transformation mechanisms but also provide innovative directions for industrial applications, including functional food development and natural product synthesis.

## 6. Conclusions and Perspectives

This review provides a comprehensive analysis of the pivotal role of enzymatic biotransformation in enhancing the value of phenolic acids within the food industry. Research indicates that enzymatic biotransformation effectively converts phenolic acids through the synergistic action of multiple pathways, including decarboxylation, reduction, and hydrolysis. The high catalytic efficiency of key enzymes offers robust support for sustainable manufacturing paradigms in food processing. However, this field still faces significant challenges, such as the need for improved catalytic efficiency, the relatively limited diversity of available enzymes, and an insufficiently comprehensive understanding of microbial metabolic networks at the systems level. To address these challenges, we propose multifaceted innovation and development strategies. These encompass enhancing the molecular performance of enzymes, extensively exploring novel enzyme resources, and leveraging synthetic biology to augment enzyme functionality. Future studies should focus on accelerating the translation of phenolic acid biotransformation technologies from laboratory innovation to industrial application by implementing these strategies.

Furthermore, through an analysis of phenolic acid biotransformation across diverse food matrices, this review reveals differential characteristics in phenolic acid composition and their transformation pathways within distinct food systems. These findings not only deepen the theoretical understanding of phenolic acid biotransformation mechanisms but also illuminate innovative directions for industrial applications, including functional food development and natural product synthesis. Consequently, this work holds substantial significance for both theoretical advancement and practical implementation.

## Figures and Tables

**Table 2:** Applications of key enzymes for phenolic acid biotransformation in fruits.

Enzyme	Original Phenolics	Transformed Products	Microorganism	Food Matrix	Role	Reference
-	Caffeic acid	Chlorogenic acid	*Lactobacillus plantarum* NCU137	Wolfberry juice	Enhances antioxidant activity	[91]
β-Glucosidase	Caffeic acid	Vanillic acid and *p*-Coumaric acid	*Lactobacillus plantarum* and *Lactobacillus acidophilus*	Strawberry juice	Enhances antioxidant activity	[156]
-	Caffeic acid and *p*-coumaric acid	Dihydrocaffeic acid and 4-ethylphenol	*Lactobacillus plantarum*	*Prunus avium* L.	Enhance flavors	[150]
DbPAD	Ferulic acid and *p*-coumaric acid	Vinyl and derivatives	*Saccharomyces cerevisiae* BY4722	Wine	Produces a unique aroma	[157]
SDPAD	hydroxycinnamic acid	4-vinyl derivatives	*Saccharomyces cerevisiae*	Blueberry wine	Enhances color stability of blueberry wines	[148]
PADC, PDC	*p*-coumaric acid	Vinyl derivatives	*Saccharomyces cerevisiae* and *Lactobacillus plantarum*	Wine	Influence on wine aroma	[147]
padC and bglB	-	Gallic acid	*Lactobacillus plantarum* T7	Mango	Gives new nutrients and flavors	[158]
reLPPAD and dLPPAD	Hydroxycinnamic acids	4-vinyl derivatives	*Pichia pastoris* GS115	*Aronia melanocarpa*	Maintains color stability and enhances the sensory evaluation of the product	[159]
-	Protocatechuic acid	Catechin	*Lactiplantibacillus plantarum* ATCC 14917 and *Limosilactobacillus fermentum* YL-11	Lychee	Enhanced nutritional and flavor properties	[160]
-	3,5-di-O-caffeoylquinic acid	Shikimic acid	*Lactobaccilus paracei*, *Lactobacillus casei*, *Lactobacillus delbrueckii* subsp. *Bifidobacterium animalis* subsp., and *Lactobacillus fermentum*	Black mulberries	Enhances antioxidant activity	[161]

- Indicates no specific enzyme or original phenolics.

**Table 3:** Applications of key enzymes for phenolic acid biotransformation in other foods.

Enzyme	Original Phenolics	Transformed Products	Microorganism	Food Matrix	Role	References
Lp_0796, Est_1092	Ferulic acid and caffeic acid	Dihydroferulic acid and dihydrocaffeic acid	*Lactobacillus plantarum* TMW1.460	Broccoli	Altering the sensory properties of food	[135]
-	Hydroxycinnamic acids	Dihydro, 4-vinyl, and 4-ethyl derivatives	*Lactobacillus plantarum* TMW1.460 and *Furfurilactobacillus milii* FUA3583	Canola meal (CM)	Increases antimicrobial activity	[166]
NCOMT	Caffeic acid	Ferulic acid	-	*Azadirachta indica*	Enhanced pharmacological activity	[168]
NlePAD	Sinapic acid	Canolol	*Neolentinus lepideus* BRFM15	Rapeseed meal (RSM)	Possesses antioxidant and anti-inflammatory activity	[169]
padC and bglB	-	Protocatechuic acid	*Lactobacillus plantarum* T7	Cress	Gives new nutrients and flavors	[158]
-	Tannic acid	Syringic acid	*Lactobacillus plantarum* InaCC B1002	Bitter gourd	Possesses antidiabetic activity	[170,171]
Tannase	Catechin gallate	Gallic acid	*Aspergillus niger* PW-2	Steamed green tea	Developing a unique sensory profile	[172]
β-Glucosidase, cellulase, and esterase	Epicatechin gallate and epigallocatechin gallate	Epicatechin and epigallocatechin	*Saccharomyces cerevisiae* Y-01, *Wickerhamomyces anomalus* ZX-1, *Lacticaseibacillus paracasei* SJ-2 and *Komagataeibacter oboediens* CGMCC 22548	Kombucha	Enhances antioxidant activity	[173,174]
β-glucosidase and esterase	-	Gallic acid	*Bacillus subtilis* CS90	*Cheonggukjang*	Improvement of antioxidant capacity	[175]
-	Hydroxycinnamates	3-(3-hydroxyphenyl)propionic acid	-	Bell pepper	Potential for cardiovascular disease protection	[176]

- Indicates no specific enzyme, original phenolics or Microorganism.

## Data Availability

No new data were created or analyzed in this study. Data sharing is not applicable to this article.
